# Biomechanism and exercise effect of fitness walking using twin walking sticks

**DOI:** 10.1093/geroni/igab046.3244

**Published:** 2021-12-17

**Authors:** Ken Yamauchi, Tsutomu Ichikawa, Akira Ogita, Hironori Yoshida, Hiromichi Hasagawa, Hiroshi Matsui, Shota kagawa

**Affiliations:** 1 Keio University, Yokohama, Kanagawa, Japan; 2 Department of Early Childhood Education and Care, Matsuyama, Ehime, Japan; 3 Osaka City University, Osaka, Osaka, Japan; 4 Liberal Arts Education Center, Ashikaga, Tochigi, Japan; 5 Fitness center, Nagakute, Aichi, Japan; 6 Kyoto Medical Center, Kyoto, Kyoto, Japan; 7 Makuhari Human Care, Chiba, Chiba, Japan

## Abstract

In Japan, walking poles with pairs of sticks developed exclusively for fitness walking have been designed. A new concept of walking style (WS) has been conceived using these walking sticks to “effectively” walk around the city, comprehensive sports parks, or at rehabilitation hospitals. Stick manufacturers are promoting its health benefits; however, evidence supporting these claims is lacking. Hence, this study aimed to measure the influence of walking sticks and evaluate the exercise effect based on functional physical fitness related to WS characteristics. The participants were 12 WS instructors. They engaged in WS at a comfortable speed after walking normally at the same speed (WN) for ∼5 m (seven times), followed by WS again. The walking speed, step length, stride width, walk ratio, one-leg support time, and trajectory of the center of gravity (CG) (in the horizontal and vertical directions of one walking cycle) calculated from the whole-body skeleton model were analyzed. The gait of WS increased the step length, step width, and walking ratio as compared with that of WN (p<0.05). WS likely reduce cadence and one-leg support time (p<0.05). The CG locus in the left-right direction showed no significant differences between WS and WN. The maximum value of the CG locus in the vertical direction was high in WS (p<0.05). WS can be used as a navigation training tool that improves a walker's exercise efficiency and left-right leg coordination, thereby improving walking posture. This may help reduce the anxiety due to injuries and pain that may occur while walking.

